# Curdlan–Chitosan Electrospun Fibers as Potential Scaffolds for Bone Regeneration

**DOI:** 10.3390/polym13040526

**Published:** 2021-02-10

**Authors:** Clément Toullec, Jean Le Bideau, Valerie Geoffroy, Boris Halgand, Nela Buchtova, Rodolfo Molina-Peña, Emmanuel Garcion, Sylvie Avril, Laurence Sindji, Admire Dube, Frank Boury, Christine Jérôme

**Affiliations:** 1 CRCINA, SFR ICAT, University Angers, Université de Nantes, Inserm, F-49000 Angers, France; clement.toullec@etud.univ-angers.fr (C.T.); nela.buchtova@gmail.com (N.B.); rodompe@gmail.com (R.M.-P.); emmanuel.garcion@univ-angers.fr (E.G.); sylvie.avril@univ-angers.fr (S.A.); laurence.sindji@univ-angers.fr (L.S.); 2 Center for Education and Research on Macromolecules (CERM), CESAM-UR, University of Liège, B-4000 Liège, Belgium; 3 Université de Nantes, CNRS, Institut des Matériaux Jean Rouxel, IMN, F-44000 Nantes, France; jean.lebideau@cnrs-imn.fr; 4 INSERM, UMR 1229, RMeS, Regenerative Medicine and Skeleton, ONIRIS, Université de Nantes, F-44042 Nantes, France; Valerie.Geoffroy@univ-nantes.fr (V.G.); boris.halgand@univ-nantes.fr (B.H.); 5 UFR Odontologie, Université de Nantes, F-44042 Nantes, France; 6 CHU Nantes, PHU4 OTONN, F-44093 Nantes, France; 7 School of Pharmacy, University of the Western Cape, Bellville 7535, South Africa; adube@uwc.ac.za

**Keywords:** curdlan, chitosan, electrospinning, regenerative medicine, tissue engineering

## Abstract

Polysaccharides have received a lot of attention in biomedical research for their high potential as scaffolds owing to their unique biological properties. Fibrillar scaffolds made of chitosan demonstrated high promise in tissue engineering, especially for skin. As far as bone regeneration is concerned, curdlan (1,3-β-glucan) is particularly interesting as it enhances bone growth by helping mesenchymal stem cell adhesion, by favoring their differentiation into osteoblasts and by limiting the osteoclastic activity. Therefore, we aim to combine both chitosan and curdlan polysaccharides in a new scaffold for bone regeneration. For that purpose, curdlan was electrospun as a blend with chitosan into a fibrillar scaffold. We show that this novel scaffold is biodegradable (8% at two weeks), exhibits a good swelling behavior (350%) and is non-cytotoxic in vitro. In addition, the benefit of incorporating curdlan in the scaffold was demonstrated in a scratch assay that evidences the ability of curdlan to express its immunomodulatory properties by enhancing cell migration. Thus, these innovative electrospun curdlan–chitosan scaffolds show great potential for bone tissue engineering.

## 1. Introduction

The loss of bone tissue represents a high socio-economical cost, and bone is the second most transplanted tissue after blood [1,2]. Bone grafts have been used in the management of traumatic injuries, genetic bone impairment and bone cancer. However, the current bone graft methods present limitations, such as lack of availability, low integration and the risk of infections, whether they are autologous, allogenic, or xenogeneic. Autologous bone graft remains the gold standard for bone regeneration and consists of taking bone tissue from one part of the body to graft it onto the defect. This can cause secondary adverse effects such as fragilization, inflammation and infection at the site of harvesting, as well as pain and morbidity [3]. Therefore, new approaches have to be explored. Tissue engineering and regenerative medicine are promising strategies for bone reconstruction. Their objective is to induce the functional regeneration of bone through a synergetic combination of scaffolds, cells and bioactive proteins.

A way to regenerate bone tissue would be through the production of a biomaterial [4,5] which mimics the extracellular matrix (ECM), making scaffolds composed of biodegradable fibers, with a strong potential to repair bone defects. These scaffolds are designed to present characteristics similar to the ECM and can provide support for the growth of new cells, specifically osteoblasts differentiated from autologous mesenchymal stem cells, favoring their adhesion, proliferation and differentiation. These scaffolds must also be able to be degraded to leave only regenerated bone tissue.

Amongst the biopolymers approved by regulation authorities [6] and produced with clinical Good Manufacturing Process (cGMP) conditions at a large scale, chitosan is very attractive. It is a linear, nontoxic, semi-crystalline [7], biocompatible [8] and biodegradable [9] polysaccharide. Chitosan is not present in nature but is easily derived from chitin, a natural polysaccharide found in the shell of shellfish and produced by some fungi [10]. Chitin is the second most common polysaccharide after cellulose [7]. Chitosan is produced from chitin through a reaction of partial deacetylation [11]. Chitosan is preferred over chitin because chitin is extremely difficult to dissolve [12], reducing its processability. Chitosan, however, is highly soluble in acidic solution due to its amine groups [13]. Chitosan presents a great number of characteristics that make it an ideal candidate for biomedical applications and the development of scaffolds for cellular regeneration. It has antibacterial [14], antifungal [15], mucoadhesive [16] and hemostatic [17] activities. Several of these characteristics come from the positive charge presented by chitosan due to the protonation of its amine functions [12]. One of chitosan’s most important characteristics for biomedical applications is its biodegradability, as it degrades into non-toxic residues in biological media [9]. Chitosan can be transformed into fibrous scaffolds by electrospinning, after being dissolved in an acidic solution allowing the apparition of positive charges on its amine groups [18]. The use of such electrospun chitosan is a subject drawing a lot of attention from researchers for its antibacterial properties [19], for wound healing [20], for the removal of dangerous metal ions [21] and for bone regeneration [22,23,24,25].

Another attractive biopolymer is 1,3-β-glucan, known as curdlan, a linear polysaccharide first observed in bacteria [26] and able to be sourced biologically [27]. It presents a chain of glucose units linked by β (1→3) linkages. Curdlan is biocompatible and has been approved for human use by the American Food and Drug Administration (FDA) [28]. Its commercially accessible version is produced by *Alcaligenes faecalis* [26]. Due to its β(1→3) linkage, curdlan presents a helicoidal structure and can form (i) reversibly at room temperature a gel presenting a mixture of single helixes and loosely tight intertwined triple helixes, as well as (ii) irreversibly at higher temperatures a gel presenting more condensed rod-like triple helixes. It is used as a thickener and fat substitute in the food industry [29]. It is also known to cause inflammation and to reactivate the immune system because of its immunomodulating properties [30], and it can be used in treatments against tuberculosis [31], HIV [32] or cancer [33].

In the field of bone regeneration, curdlan is a particularly interesting material as it enhances bone growth by helping mesenchymal stem cell adhesion onto the scaffold and by favoring their differentiation into osteoblasts [34]. It also has an inhibiting effect on osteoclastogenesis through its interaction with the dectin-1 receptor [35,36], leading to a further increase in the speed of the regeneration of bone tissue.

To create polymer scaffolds, several techniques exist, such as phase separation or freeze-drying. Curdlan and chitosan have been combined, for example, by Przekora et al., who formed curdlan/chitosan/hydroxyapatite scaffolds through a gelation technique [16,37]. However, those hybrid materials are problematic to handle and to formulate due to poor biopolymer solubilization; the use of porogeneous components and drying steps complicate their development and scale-up. In this study, we have chosen to focus on electrospinning, which generates fibrillar scaffolds mimicking the structure of the Extra Cellular Matrix (ECM) [38,39]. The electrospinning technique has the versatility to process a wide range of materials in order to produce scaffolds with the required morphology and porosity, including fibers with diameters from a few micrometers down to the hundred nanometer range [40]. Electrical charges are used to draw, by means of a syringe pump, fine fibers and create, with a collector, a nonwoven fibrous mat with high porosity and surface areas able to adsorb proteins and binding sites to cell membrane receptors. Another important feature of this technique is that the electrospun fibers can be further functionalized via the incorporation of bioactive species. Several scaffolds for bone tissue engineering applications have already been prepared by electrospinning using biocompatible polymers such as poly(lactic-co-glycolic acid) (PLGA) [41], polycaprolactone [2], silk fibroin [42] and chitosan [11]. Electrospinning of chitosan is known to combine stability and high porosity and mimic the extracellular matrix [43]. Electrospun chitosan exhibiting this fibrillar morphology has demonstrated better efficiency as scaffolds for skin regeneration than porous sponges or films [44]. Even the electrospun chitosan mats are promising; the chemical modification is in some cases required to endow novel types of functions that are useful in biomedical applications. In this context, producing composite curdlan–chitosan scaffolds appears to be a very appealing alternative approach, combining the mechanical properties of both biopolymers and taking advantage of the immunomodulation properties of the curdlan [30]. Basha et al. managed to electrospin curdlan with poly(vinyl alcohol) (PVA) in a solution of formic acid [45,46]. Nevertheless, PVA is a non-biodegradable material and, moreover, has no biological activity. In the present study, a blend of chitosan and curdlan was electrospun to form a composite scaffold composed of submicronic fibers. To the best of our knowledge, this is the first example of the use of this method to form curdlan–chitosan fibers. This novel method will allow the fabrication of a highly porous, highly stable implant, combining the mechanical and biological properties of curdlan and chitosan. We evaluated the physical characteristics of the scaffold as well as its cytocompatibility and its ability to enhance cell spreading.

## 2. Materials and Methods

### 2.1. Materials

Curdlan, polyethylene oxide (PEO, 2MDa), glycofurol, sodium chloride, sodium hydroxide, dimethyl sulfoxide, tris base (Trizma), glycine, formic acid, acetic acid and 37% hydrochloric acid were purchased from Sigma (Saint Quentin Fallavier, France). Chitosan (deacetylation degree: 95%, viscosity ≤ 7 mPas) was purchased from Heppe Medical Chitosan (Halle, Germany). Ultrapure water was obtained from a Milli-Q Advantage A10 System (Millipore, Paris, France)

Dulbecco’s phosphate-buffered saline (PBS, Biowhittaker^®^) was purchased from Lonza (Verviers, Belgium), bovine serum albumin fraction V was purchased from Roche Diagnostics GmbH (fetal bovine serum (FBS), Mannheim, Germany) and Dulbecco’s Modified Eagle’s Medium (DMEM, Gibco^®^) was purchased from Thermo Fisher Scientific (Villebon sur Yvette, France).

The 3-(4,5-dimethylthiazol-2-yl)-5-(3-carboxymethoxyphenyl)-2-(4-sulfophenyl)-2H-tetrazolium)(MTS) assay was purchased from Promega (Madison, WI, USA) as CellTiter 96^®^ AQueous Non-Radioactive Cell Proliferation Assay.

All materials were used without further treatment or purification unless otherwise stated.

### 2.2. Electrospinning of Chitosan and Curdlan–Chitosan into Fibrillar Scaffolds

A solution of curdlan was prepared by dissolution overnight of 300 mg of curdlan into 4.5 mL of 85% formic acid. Separately, 600 mg of chitosan was dissolved in 8 mL of 4.5% acetic acid. A total of 86 mg of PEO was dissolved in 3 mL of MilliQ water. The three optically transparent solutions were then gently mixed under magnetic stirring at 100 rpm for an hour, resulting in a homogeneous, white, opaque mixture that is used for electrospinning. In this mixture, the resulting weight ratio of curdlan:chitosan is 1:2.

This solution was then placed into a 10 mL soft inject syringe with a 1.5 inch needle. It was left standing for another hour for degassing. Between the needle of the syringe and an aluminum foil collector located 17 cm from the tip of the needle, an electrical tension of 20 kV is applied for 30 min, the mixture being electrospun at a flowrate of 0.6 mL/h.

The neat chitosan scaffold was made in a similar way, with 400 mg of chitosan in 6.8 mL of 4.5% acetic acid solution, 60 mg of PEO in 2.8 mL of MilliQ water, an electrical tension of 30 kV and a flowrate of 0.78 mL/h. All other electrospinning parameters were maintained identical as for the curdlan–chitosan scaffold.

The scaffolds collected on the aluminum foil were stabilized by dipping into absolute ethanol for 30 s and then into a sterile solution of NaOH at a 1 M concentration for 30 s. They were then immerged in sterile water for 15 min, dipped in a 70% ethanol solution for 30 s and then plunged into sterile water for 15 min, twice more.

### 2.3. Characterization of the Fibrillar Scaffolds

The morphology of the fibrillar scaffolds was characterized by Scanning Electron Microscopy (SEM) using JEOL microscope model JSM-6310F or JSM-840A with an acceleration voltage of 3 keV and 20 keV, respectively. The fibers were sputter coated with platinum before observation. The diameter of the fibers was determined by measuring 100 randomly selected fibers using the ImageJ software.

The composition of the scaffolds was also studied using Fourier Transform Infrared spectroscopy (FTIR) (Nicolet iS5^®^ spectrometer, Thermo Fisher Scientific) in the spectral range of 4000–510 cm^−1^ with a spectral resolution of 4 cm^−1^. FTIR spectra have been recorded in Attenuated Total Reflectance (ATR) mode.

### 2.4. Swelling and Degradation Experiments

For the swelling study, 3 × 1 cm of stabilized and dried chitosan or curdlan–chitosan scaffolds were weighed and immersed in PBS and then placed at 37 °C in a thermostatically controlled incubator. Scaffolds were removed at defined time intervals. The excess of PBS at the surface was blotted using filter paper and the swelled scaffolds were weighted. The swelling ratio (in%) was calculated through the weight ratio between the dry and wet scaffold as follows:

$$\mathrm{Swelling}\;\mathrm{ratio}=\;\frac{\mathrm{mass}\;\mathrm{of}\;\mathrm{swollen}\;\mathrm{scaffold}-\mathrm{mass}\;\mathrm{of}\;\mathrm{dry}\;\mathrm{scaffold}}{\mathrm{mass}\;\mathrm{of}\;\mathrm{dry}\;\mathrm{scaffold}}*\;100$$

For the enzymatically assisted hydrolytic degradation study, 3 × 1 cm of stabilized and dried chitosan or curdlan–chitosan scaffolds were weighed and immersed in 2 mL of a 0.05 M Tris-HCl buffer solution (pH 7.4) supplemented with 0.15 M NaCl, 20 μg/mL lysozyme and 1 mg/mL bovine serum albumin (BSA). Scaffolds were removed at defined time intervals. The samples were rinsed in sterile water and dried under laminar flow air overnight. The mass loss (in%) was determined by weighting the samples before and after the incubation as follows:

$$\mathrm{mass}\;\mathrm{loss}=\;\frac{\mathrm{mass}\;\mathrm{before}\;\mathrm{degradation}-\mathrm{mass}\;\mathrm{after}\;\mathrm{degradation}}{\mathrm{mass}\;\mathrm{after}\;\mathrm{degradation}}*100$$

### 2.5. Cytotoxicity Test

The cytotoxicity evaluation of the scaffolds was performed with murine L929 fibroblasts (ECACC General Collection N°85011425, ATCC^®^ CCL-1™) by MTS assay. The cells were seeded at 10,000 cells/cm^2^ into 24-well cell culture plates and cultured for 24 h with 1 mL of culture media (DMEM + 10% FBS + 1% penicillin/streptomycin). A piece of folded scaffold (3 × 1 cm), either chitosan or curdlan–chitosan, was added to each well and cells were cultured for additional 24, 48 or 72 h. At the end of the culture time, the culture media were replaced by 500 μL of media with MTS (20 μL of MTS preparation for 100 μL of media) and incubated for 3 h. The culture medium was then collected and placed into a 96-well plate at 100 μL per well. Absorbance was measured at 490 nm using a multimode plate reader (Multiskan Ascent, Labsystems, Les Ulis, France). Metabolic activity was expressed as percent of control.

### 2.6. Cell Spreading Adhesion Test

Stabilized chitosan and curdlan–chitosan scaffolds were placed in each well of a 6-well tissue culture plate. L929 murine fibroblasts were seeded at a concentration of 10,000 cells per cm^2^. Samples were incubated at 37 °C and 5% CO_2_ for 72 h in culture media (DMEM + 10% FBS + 1% penicillin/streptomycin). The medium was changed after 48 h. Then, the cell-seeded scaffolds were removed, rinsed twice with PBS to remove the nonadherent cells, subsequently fixed with 2.5% glutaraldehyde for 1.5 h, rinsed with PBS and stained with a 1% solution of osmium tetroxide during 45 min. The samples were rinsed in MilliQ water, dehydrated using alcohol gradient (50% ethanol for 15 min then 70%, 95% and 100% twice for 30 min), desiccated with hexamethyldisilazane (HMDS) and air dried overnight. They were sputter coated with platinum and observed under a SEM (JSM 6310F, JEOL, Paris, France) at 3 keV.

### 2.7. Scratch Assay

L929 fibroblast cells were seeded into a 6-well plate and grown to a sub-confluent (80–90%) monolayer. A scratch was gently made in the center of the cell monolayer using a sterile 20 μL pipette tip. The debris were washed away with PBS. A 2 mg non-stabilized scaffold sample was dissolved in 100 µl of sterile water and mixed with 1.9 mL of culture medium without serum. This preparation was added to the wells. After 24 h, the cells were washed with PBS and stained with a trypan blue solution for better contrast. Representative images from each well of the scratched area were photographed using an optical microscope and a built-in camera (AxioCam^®^ ICm 1, Zeiss, Jena, Germany) to observe the migration of the cells.

### 2.8. Statistical Analysis

Data are presented as the mean value ± standard deviation (SD) of at least three experiments (n ≥ 3). Statistical significance was determined using two-way ANOVA followed by Bonferroni’s post-test for multiple group comparison. Statistical significance was set at *p* < 0.05. The statistical analysis program used was Prism 8 (version 8.4.3).

## 3. Results and Discussion

### 3.1. Scaffolds Preparation and Morphology

**Figure 1:**
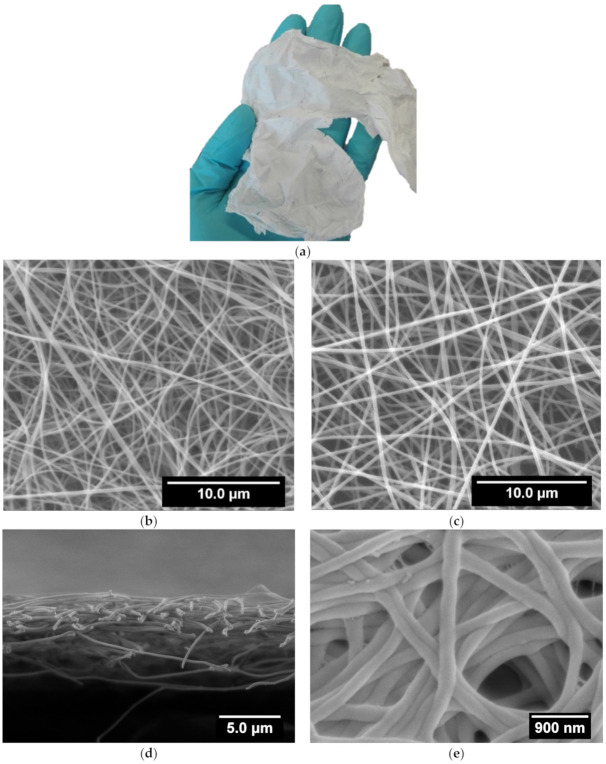
(**a**) Photography of the macroscopic aspect of the curdlan–chitosan scaffold as removed from the collector and SEM images of (**b**) electrospun chitosan membrane (Acceleration Voltage (AV) 20 keV), (**c**) electrospun curdlan–chitosan membrane (AV 20 keV), (**d**) cross section of electrospun curdlan–chitosan membrane (AV 3 keV), (**e**) electrospun curdlan–chitosan membrane after stabilization (AV 3 keV).

Curdlan is insoluble in water and in most organic solvents, though it is soluble in dilute alkali (0.25 M NaOH) and dimethyl sulfoxide. Formic acid was selected from among its few solvents [29] as an acidic solution most appropriate for electrospinning and blending with the chitosan solution. Indeed, chitosan is soluble in acetic acid due to protonation of the amino groups leading to positively charged polyelectrolyte. As reported previously, for chitosan to be electrospun from such an acidic solution requires the presence of PEO to promote chain entanglement of the chitosan strands by forming hydrogen bonds between the ether oxygen of PEO and the amino hydrogen of chitosan [47]. Indeed, the strong electrostatic repulsion between the positively charged deacetylated groups on the chitosan backbone can prevent a sufficient entanglement of the chains to form fibers [48]. Therefore, PEO was also added to the chitosan/curdlan mixture used for electrospinning. By replacing 1/3 of the chitosan by curdlan in the electrospinning mixture, and adjusting the mixture composition to reach similar viscosities, the deposition of a regular fiber mat is efficiently obtained on the collector in operating conditions adapted to the blend. Optimization of the solution flow rate and electrical tension was necessary to compensate the screening of charges of curdlan and viscoelastic variations of the curdlan/chitosan mixture as compared to chitosan without curdlan in order to reach defect-free fibers with the blend. These mats can be easily detached from the Al foil collector as a free-standing membrane (Figure 1a) that can be handled without breaking.

Figure 1b,c shows the morphology of the electrospun chitosan and curdlan–chitosan blend membranes, respectively. In both cases, well-defined fibers are observed without beaded defects, evidencing that the applied electrospinning conditions were appropriate [18]. It is worth mentioning that after 30 min of electrospinning the dry collected chitosan/curdlan scaffold is 5 µm thick, as can be seen in Figure 1d.

**Figure 2:**
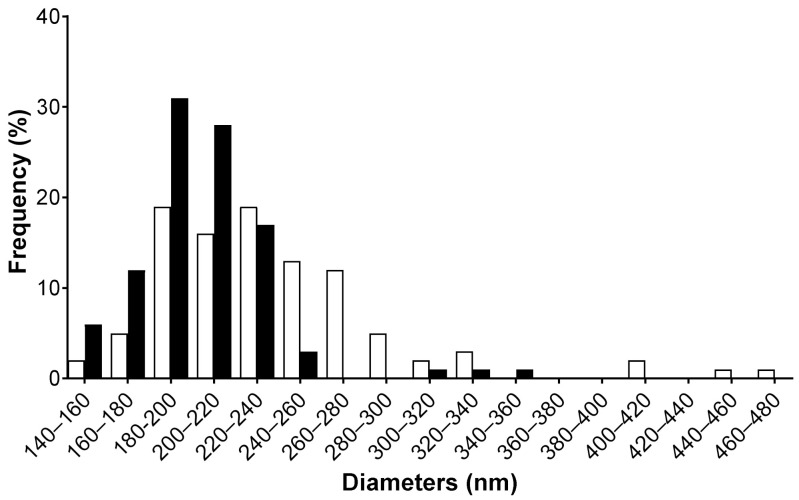
Size distribution of the fiber diameter for chitosan membrane in white and curdlan–chitosan membrane in black measured by image analysis with ImageJ on 100 randomly selected fibers.

Images analysis of such SEM pictures allows us to determine the fiber size distribution, as reported in Figure 2. It evidences that the diameter of the chitosan fibers has an even distribution with a higher number of large diameter fibers than for the curdlan–chitosan fibers, which follow a standard distribution with few fibers falling out of it at higher diameters. From these distributions, average fiber diameters of 205 ± 40 nm and 238 ± 80 nm are obtained for curdlan/chitosan and chitosan membranes, respectively. The slight decrease in size of the fibers with curdlan might be due to the increased acidity of the solution containing curdlan due to the presence of formic acid, increasing the polarity of the solution and therefore the stretching of the polymer jet [46,49]. A slight change in viscosity due to the lower solubility of curdlan could also explain the difference in diameter between the two scaffolds. The diameter of the obtained fibers is appropriate for bioengineering applications as the purpose is to mimic the fibrils of the extracellular matrix, whose dimensions range from 10 to 300 nm in diameter [50].

Nevertheless, before using these fibrillar membranes in aqueous media for cells culture, they have to follow a stabilization process to avoid their dissolution. Indeed, the chitosan is highly protonated in the electrospun scaffold, making it soluble in water. To prevent the scaffolds from dissolving in water, it is necessary to neutralize the positive charges on the polysaccharide by bringing it in contact with a strong basic solution, followed by washing with water and ethanol for sterilization. After this stabilization treatment, the fibers appeared flattened and agglomerated, as can be seen in Figure 1e. This results from the decrease in the electrostatic repulsion as a consequence of the chitosan neutralization. However, the average diameter of such stabilized curdlan–chitosan fibers is 216 ± 60 nm, which remains very close to the diameter of the fibers before stabilization.

### 3.2. Scaffolds Chemical Composition

The chemical structure of chitosan (β-1,4-glucosamine) and curdlan (β-1,3-glucan) is similar. Therefore, both attenuated total reflection—Fourier Transformed Infra-red (ATR-FTIR) spectra show several similarities, such as -OH vibrations at around 3300 cm^−1^, -CH stretching at around 2900 cm^−1^, C-O-C asymmetric stretching at 1153 cm^−1^, and C-O stretching at 1066 and 1028 cm^−1^ [51]. However, some bands are more specific to a given polysaccharide: the N-H bending band exists only for chitosan, and a non-attributed band at 1530 cm^−1^ is present only in the curdlan spectrum.

**Figure 3:**
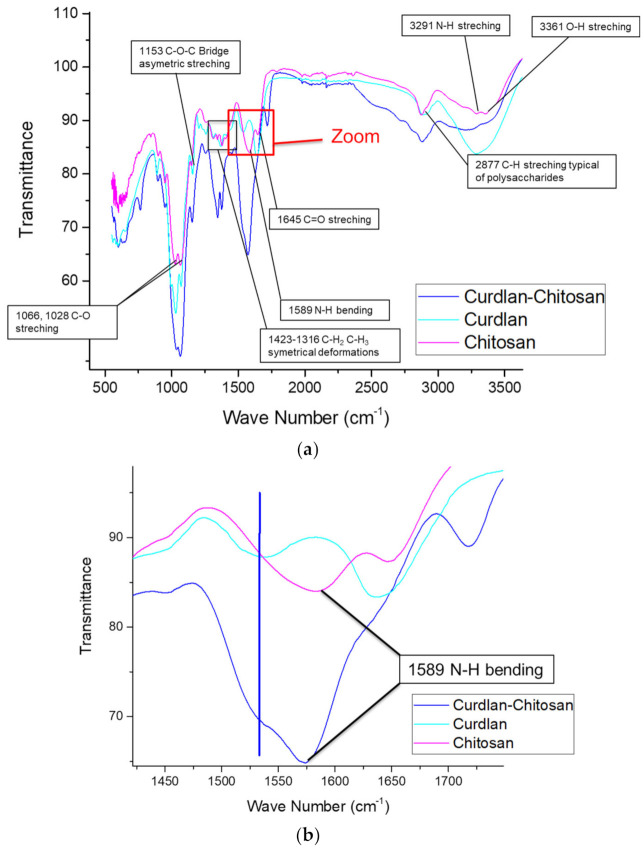
ATR-FTIR spectra of curdlan and chitosan starting polymers and of the curdlan–chitosan fibers. (**a**) The full spectrum with peaks attribution [51]. (**b**) Zoom on the 1400 to 1700 cm^−1^ spectral range to identify characteristic peaks of both curdlan and chitosan of the blend spectrum (blue line denotes the 1530 cm^−1^ band specific to curdlan).

Figure 3 describes the complete ATR-FTIR spectra of curdlan, chitosan, and the curdlan–chitosan scaffold (A), as well as a zoom of the region between 1425 cm^−1^ and 1700 cm^−1^ (B). The zoom showed us the presence of both identifying peaks in the curdlan–chitosan scaffold, demonstrating the presence of both non-denatured polysaccharides in the fibers.

### 3.3. Scaffolds Swelling and Degradation

**Figure 4:**
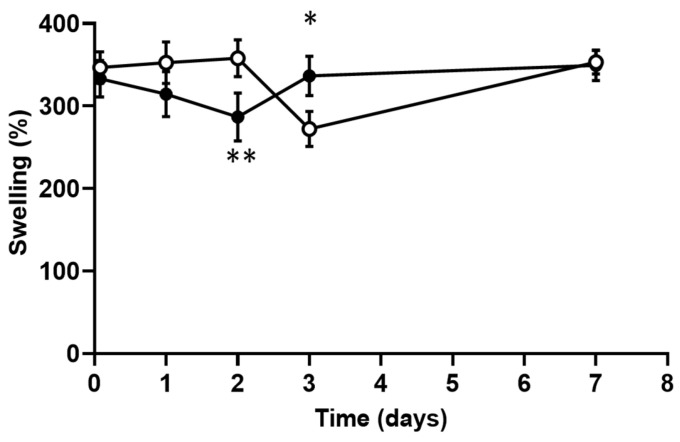
Swelling profile of the chitosan scaffolds (white dots) and curdlan–chitosan scaffolds (black dots) over time (* *p* < 0.05 et ** *p* < 0.01).

The ability of a scaffold to swell is directly related to the ability of cells to infiltrate and attach themselves onto the scaffold. High swelling means greater opening of the fibers and a higher surface area for cell adhesion. Figure 4 shows the high swelling for the chitosan and curdlan–chitosan membranes (between 300% and 350%). This swelling is already maximal after 2 h immersion in PBS (first measurement) and remains quite constant over time in a period of 7 days.

**Figure 5:**
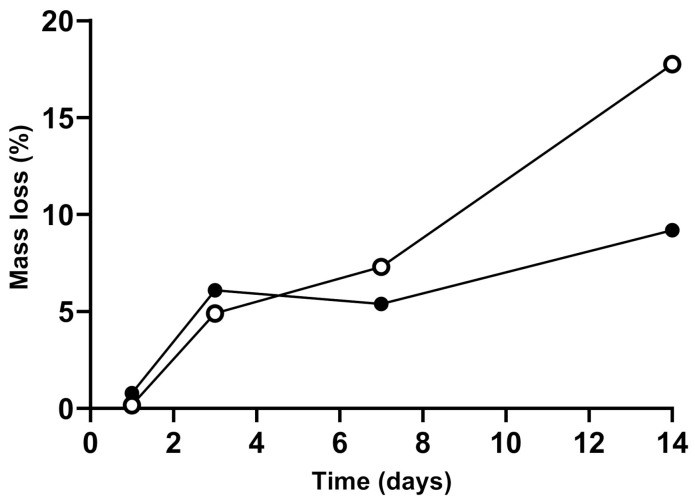
Degradation of the chitosan scaffolds (white) and curdlan–chitosan scaffolds (black) over time.

As shown in Figure 5, the enzymatic degradation of the chitosan scaffold was regular over time, with a loss of 15% of its original mass after 12 days. The degradation of the curdlan–chitosan scaffold was slower than the chitosan scaffold, losing 8.5% of its original mass over 12 days. This shows that the presence of curdlan decreased the degradation rate of the scaffold owing to curdlan’s resistance to hydrolysis and hydrolases [52]. This will allow the scaffold to maintain its mechanical properties for a longer period of time.

### 3.4. In Vitro Scaffold Cytotoxicity Assays

**Figure 6:**
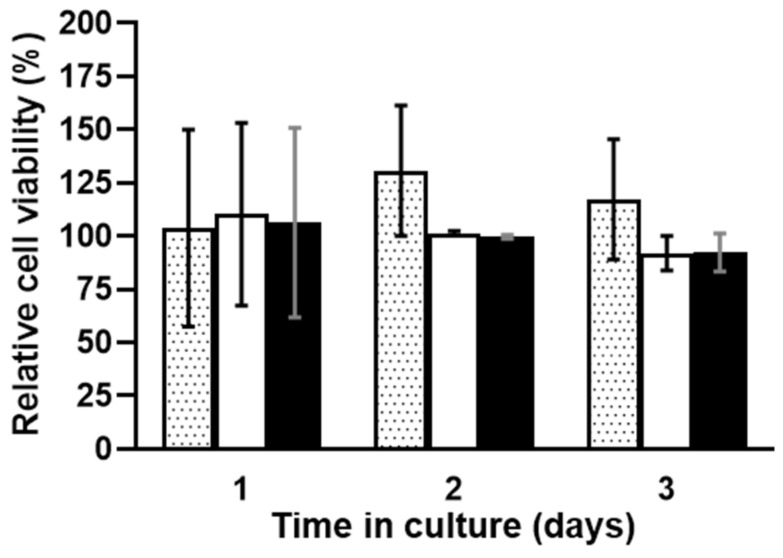
Metabolic activity over time of L929 mice fibroblast cells, control assays (dotted bars) and in the presence of pure chitosan scaffold (white bars) and curdlan–chitosan scaffold (black bars). Cell viability is expressed as % of the control evaluated at d0 (cells cultured in Dulbecco’s Modified Eagle’s Medium (DMEM) with 10% FBS). Values are presented as mean ± SEM.

Figure 6 shows the metabolic activity over time of the L929 mice fibroblast cells in the presence of chitosan and curdlan–chitosan scaffolds. ANOVA statistical study shows no significant effect of time or the presence of either scaffold on cell viability. It is a good in vitro indicator for potential bioengineering applications.

### 3.5. Cell Migration Study

**Figure 7:**
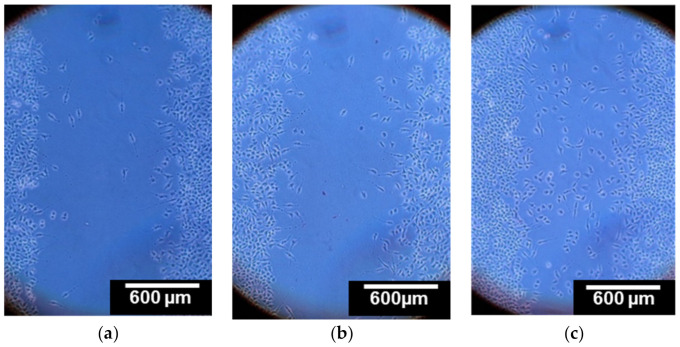
Scratch assay with L929 mice fibroblast cells after 24 h in serum-free culture medium without any scaffold under microscope at 10× magnification, (**a**) with dissolved non-stabilized chitosan scaffold, (**b**) and with curdlan–chitosan scaffold (**c**). The images are representative of 3 independent experiments.

A scratch assay was performed and showed that cells that were in contact with dissolved curdlan have a greater mobility as compared to those that were not in contact with curdlan. As can be seen in Figure 7, cells in contact with the dissolved chitosan scaffold did not move into the gap faster in comparison to the control. Cells in contact with the curdlan/chitosan spread more effectively, showing a greater mobility of the cells, which can only be due to the presence of curdlan. The ability of β-glucans such as curdlan to improve wound healing through stimulation of the biosynthesis of collagen has been reported before [53,54]. This preliminary study confirms that curdlan does not lose its biological activity after the electrospinning process.

### 3.6. Cell Spreading Study

**Figure 8:**
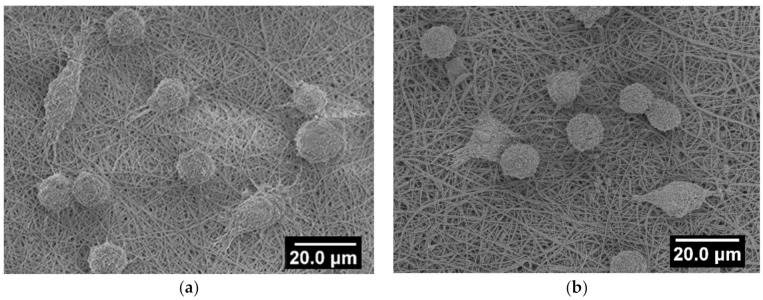
SEM images of L929 mice fibroblast cells on chitosan (**a**) and curdlan–chitosan (**b**) scaffold after 72 h of culture. The images are representative of 3 independent observations (AV 3 keV).

The ability of cells to spread on the surface of a material is essential for this material to be used for bioengineering purposes. Cell attachment and spreading on highly deacetylated chitosan are known to be low [55,56]. We expected that the ability of the curdlan to swell and to fix cells [34] would improve the cell spreading on the surface of the curdlan/chitosan scaffolds. However, the images shown in Figure 8 suggest that the addition of curdlan into the fibers has not significantly improved cell spreading. Furthermore, the cell density does not seem to be higher on the curdlan–chitosan scaffold than on the chitosan scaffold. On either scaffold, a small number of cells were spread on the surface, most of which retained a round shape suggesting poor adhesion on the material; further experiments would be needed to prove this. An additional treatment, such as adhesion cytokine (e.g., immobilizing fibronectin [56]), on the scaffold might be necessary for improving these results. Thinner fibers and higher porosity could be achieved, for which we expect a better cell spreading on the scaffolds.

## 4. Conclusions

We have successfully electrospun a novel scaffold composed of chitosan and curdlan with fibers of 200 nm in diameter. This is the first step in the development of a scaffold for which we foresee the encapsulation and controlled release of the bone morphogenic protein 2 (BMP-2) for the differentiation and proliferation of mesenchymal stem cells (MSCs) and the formation of bone tissue for regenerative medicine.

Curdlan is a material of particular interest because of its unique biological properties, having a positive effect on the proliferation of MSCs and their differentiation in osteoblasts. The curdlan–chitosan scaffolds were shown to be non-cytotoxic and able to improve cell migration due to the properties of curdlan. A number of challenges remain, such as improvement of the ability of cells to spread on the surface of the scaffold. We will also need to present evidence that these scaffolds allow MSC proliferation and differentiation toward osteoblastic lineage and to characterize cell adhesion at the material surface.

This type of scaffold could bring interesting routes to bone regeneration, as well as make use of the immunomodulator effect of curdlan in cancerology and infectiology [30].

## Data Availability

Not applicable.
